# Paediatric HIV: we are not done yet!

**DOI:** 10.1002/jia2.25643

**Published:** 2020-11-20

**Authors:** Martina Penazzato

**Affiliations:** ^1^ Global Programme on HIV, Hepatitis and STIs World Health Organization Geneva Switzerland

**Keywords:** HIV, children, antiretroviral therapy, paediatric HIV

1

There is no doubt about the exceptional progress made by the global community to date in preventing vertical transmission of HIV, halving the number of new infections in children and rapidly expanding access to antiretrovirals for pregnant women living with HIV [1]. While only a few countries have reached validation of the elimination of vertical transmission, several others are developing a vision and a path to a future where no child is born with HIV [2].

The optimism surrounding the prevention of vertical transmission has, however, promoted a false perception that reaching an AIDS‐free generation is just a matter of time. The 2019 UNAIDS estimates confirm that children continue to be left behind [1] and call on us to challenge our complacency around poorer treatment outcomes for children and adolescents: each day, about 400 children acquire HIV globally and around 270 die because of AIDS‐related deaths; a third of infants born to mothers living with HIV do not receive an HIV test by two months of age; only 53% of the 1.8 million children living with HIV receive life‐saving ART, and when they do, they have poorer virological suppression compared to adults [3].

This situation is unlikely to improve in 2020 due to what many consider a “public health earthquake,” the COVID‐19 pandemic, which is exposing health systems’ weaknesses and potentially reverting the progress made [4]. Disruption of essential services continues to be reported, including those delivered through antenatal and postnatal care, such as HIV testing and ART delivery for mothers and their children. Stock‐outs of paediatric formulations are also of concern, at a time when countries are undertaking a major shift to more optimal regimens for children [5]. Modelling scenarios show a potential 86% increase in the number of new paediatric infections due to COVID‐19‐related care disruptions, which would erase five years of hard‐won progress (J Stover, personal communication). Intensified focus and immediate action are needed to compensate for these new challenges.

## 2 AN URGENT NEED TO GO BACK TO THE BASICS AND USE NEW TOOLS

Even the most basic proven interventions are often not implemented widely. An example is the poor uptake of family based testing and index‐case testing of adults living with HIV to identify children within their households [6]. Data from several countries demonstrate that successful implementation of index‐case testing results in the identification of children with HIV that would otherwise go undiagnosed [6]. This intervention is inexpensive and relatively easy to deliver – so there should be no reason for not diagnosing the children of parents living with HIV who are already in HIV care and right in front of us.

There are also new tools that we can rely on. Multiple randomized control trials, in settings with both strong and weak laboratory systems, have demonstrated that adoption of point of care early infant diagnosis can reduce the turn‐around time for test results down to a few hours, remarkably shortening the time to treatment initiation [7, 8, 9, 10]. Moreover, we have learned that these types of innovations can be even more transformative if we deliver them in combination with other interventions, which is particularly true for infants who continue to be affected by high mortality [11]. A better package of care for infants is urgently needed, and a good step in the right direction is the regulatory approval of dolutegravir (DTG) paediatric dispersible tablets, which allows for the use of DTG from 3 kg and four weeks of life. Additional generic child‐friendly formulations of DTG are awaiting regulatory approval and will broaden access. This was possible thanks to an unprecedent level of collaboration between the drug’s innovator company, research networks, generic manufacturers, donors and key stakeholders, which has set an example for future drug development programmes.

**Figure 1:**
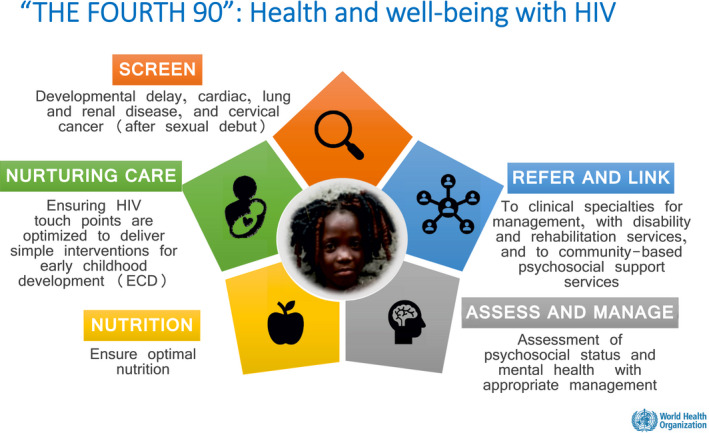
Addressing the “4th 90” and reaching children’s full potential

As better treatment is successfully implemented, an emerging issue to tackle is what can be considered the “4^th^ 90.” This would ensure that children living with HIV (CLWH) not just survive and maintain virological suppression but are given the chance to have an improved quality of life and reach their full potential (Figure 1). Reaching the fourth 90 requires having a long‐term vision for our paediatric HIV programmes, and acknowledging that screening for neurodevelopment and growth delays, promoting nurturing care and supporting mental and psychological development of children and adolescents as they age are of paramount importance. Until the fourth 90 is tackled, the job will not be done [12].

## 3 INVESTIGATING THE “HOW TO” FOR THE HIGHEST IMPACT

To achieve impact, the ways interventions are delivered are often as important as the interventions themselves. A good example comes from the Zvandiri‐Africaid programme in Zimbabwe, which demonstrated that providing HIV services to adolescents with the support of their peers can improve virological suppression and impact health outcomes [13]. This is one of the few examples of robust operational research that has tested a model tailored to deliver care to adolescents and then informed policies resulting in concrete change in multiple countries. More studies of this kind should be carried out in the future.

Strategies to optimally deliver interventions and take them to scale need to be tailored to the epidemic context and informed by well‐conducted operational research. This type of research is too often overlooked and not well funded nor supported in a systematic way. Moving forward, it will be essential to increase the capacity of those who can lead this type of research on the ground and to promote stronger collaborations to maximize expertise and impact.

## 4 PROMOTING INNOVATION TODAY SO THAT WE CAN IMPROVE CHILDREN’S OPTIONS TOMORROW

Over the past year, the global community has witnessed an unprecedent level of collaboration in research and development for Covid‐19. Trials such as the Recovery trial have included paediatric subjects from the start, and a plan to investigate and approve remdesivir in children has been designed as a simple small pharmacokinetics study where children of different ages are enrolled at the same time based on weight. This approach has the potential to accelerate the way new drugs are studied in children and is the standard we are looking for with new antiretrovirals, especially those of interest for their long‐acting potential, which could greatly impact children and adolescents’ adherence to ARVs. Research efforts surrounding COVID could and should be an inspiration as to how new treatments for HIV are studied, so that they do not continue to reach children the usual 10 years after their market authorization in adults, and that the pipeline of new technologies to deliver medicines to children [14] is fully utilized. No organization can do this alone. We can only succeed if we partner effectively and efficiently. This is the philosophy behind the Global Accelerator for Paediatric formulations (GAP‐f) [15], a newly established WHO network, which aims to accelerate investigation, development and introduction of the most needed paediatric formulations across the various stages of the product life‐cycle to make better antiretrovirals available to children and their families.

5

In order to achieve an AIDS‐free generation, we must act, research and innovate. Our path to success requires leadership, partnership and action as well as acknowledging this as a shared responsibility. Today we have a choice: let COVID‐19 stop our progress or use it as an opportunity to bounce forward and build back better. It is all up to us.

## CONFLICT OF INTERESTS

No conflict of interest to declare.

## AUTHOR CONTRIBUTIONS

MP developed the content and wrote the manuscript.

## ABBREVIATIONS

ART, Antiretroviral therapy; CLWH, Children living with HIV; DTG, Dolutegravir; GAP‐f, Global Accelerator for Paediatric formulations; POC EID, Point Of Care Early Infant Diagnosis.

## FUNDING

The author received no specific funding for this work.
